# Data sharing in the age of predictive psychiatry: an adolescent perspective

**DOI:** 10.1136/ebmental-2021-300329

**Published:** 2022-03-28

**Authors:** Gabriela Pavarini, Aleksandra Yosifova, Keying Wang, Benjamin Wilcox, Nastja Tomat, Jessica Lorimer, Lasara Kariyawasam, Leya George, Sonia Alí, Ilina Singh

**Affiliations:** 1 Department of Psychiatry, University of Oxford, Oxford, UK; 2 Wellcome Centre for Ethics and Humanities, University of Oxford, Oxford, Oxfordshire, UK; 3 Ethox Centre, Department of Population Health, University of Oxford, Oxford, UK; 4 Department of Cognitive Science and Psychology, New Bulgarian University, Sofia, Bulgaria; 5 Institute of Cognitive Neuroscience, University College London, London, UK; 6 Department of Neuroscience, University of Sheffield, Sheffield, UK; 7 Department of Philosophy, Faculty of Arts, University of Ljubljana, Ljubljana, Slovenia; 8 Department of Psychology, University of Southampton, Southampton, UK; 9 Division of Psychology & Language Sciences, University College London, London, UK; 10 Department of Psychology, University of Sussex, Brighton, UK

**Keywords:** child & adolescent psychiatry

## Abstract

**Background:**

Advances in genetics and digital phenotyping in psychiatry have given rise to testing services targeting young people, which claim to predict psychiatric outcomes before difficulties emerge. These services raise several ethical challenges surrounding data sharing and information privacy.

**Objectives:**

This study aimed to investigate young people’s interest in predictive testing for mental health challenges and their attitudes towards sharing biological, psychosocial and digital data for such purpose.

**Methods:**

Eighty UK adolescents aged 16–18 years took part in a digital role-play where they played the role of clients of a fictional predictive psychiatry company and chose what sources of personal data they wished to provide for a risk assessment. After the role-play, participants reflected on their choices during a peer-led interview.

**Findings:**

Participants saw multiple benefits in predictive testing services, but were highly selective with regard to the type of data they were willing to share. Largely due to privacy concerns, digital data sources such as social media or Google search history were less likely to be shared than psychosocial and biological data, including school grades and one’s DNA. Participants were particularly reluctant to share social media data with schools (but less so with health systems).

**Conclusions:**

Emerging predictive psychiatric services are valued by young people; however, these services must consider privacy versus utility trade-offs from the perspective of different stakeholders, including adolescents.

**Clinical implications:**

Respecting adolescents’ need for transparency, privacy and choice in the age of digital phenotyping is critical to the responsible implementation of predictive psychiatric services.

## Background

Psychiatry has traditionally relied on behavioural observations, genomics and neuroscience to draw conclusions about the aetiology of mental health challenges and build predictive risk models. However, as digital data have become increasingly ubiquitous, other data sources have emerged as relevant for early detection of psychiatric problems. These include activity data, speech features, social media interaction and typing speed, all of which can be collected via smartphones and wearables.^1 2^ Coupled with appropriate analytical methods, these multiple sources of data might be used to build models capable of predicting psychiatric outcomes for individuals.^2^

Such advances impact young people, particularly minors, who are heavy users of digital technologies. Adolescents are also likely to be key targets for early screening and early intervention programmes in mental health based on big data technologies.^2 3^ Indeed, even though research is still in its infancy, a number of companies have already started offering predictive psychiatric testing services.^4^ To mention a few examples, Social Sentinel (https://www.socialsentinel.com/) scans digital signals to detect safety and security threats in school, including self-harm; Steer (www.steer.global) offers emotional tracking for early identification of mental health challenges and 23andme (www.23andme.com) offers predictive genetic testing for Alzheimer’s disease. There is also enthusiasm for the possibility of linking/sharing data between schools and health systems to support research and intervention in adolescent mental health^5^ and for health systems and schools to use social media data to monitor mental health risk.^6^

Scientific advances in predictive mental health, alongside the increasing availability of commercial testing services, have sparked significant debate over potential ethical and social implications.^7 8^ For instance, researchers have questioned the current utility of predictive tests in psychiatry, given their limited predictive power.^9 10^ Others have pointed to the unique sensitivity of mental health data, which may not only be used for accessing health and social services but also for influencing criminal justice proceedings (eg, sentence mitigation), and might attract stigma, discrimination and forced treatment.^8^ Associated issues surrounding who owns and who should be granted access to mental health data have also been raised.^7^ In the context of minors, researchers have debated who has the right to manage a child’s risk, and raised privacy concerns regarding data sharing.^3 11 12^

Before predictive mental health services are widely implemented, it is essential to understand the extent to which young people value these services and their attitudes and preferences in relation to sharing different sources of personal data for such purpose. Empirical ethics research so far has largely focused on university students and predictive testing based on genetic information. Studies suggest that young people’s interest in learning about their genetic susceptibility for psychosis is low among non-clinical populations^13^ and high among clinical high-risk participants.^14^ Yet, high-risk participants expressed worries surrounding stigma, data privacy and potential psychological harm of genetic risk information for psychosis.^15^ Similar concerns were expressed by grandchildren of people with late-onset Alzheimer’s disease with regard to testing for a susceptibility gene.^16^ It is unknown, however, whether existing evidence related to psychosis and Alzheimer holds for attitudes to risk prediction for other psychiatric or neurological difficulties or predictions based on other types of data, especially digital.

While there has been research conducted more generally on young people’s views towards data sharing online, for example, investigating their privacy and safety attitudes,^17 18^ to our knowledge, young people’s attitudes towards digital phenotyping in mental health have only been investigated in a small scoping study with 15 (university) students in the UK.^19^ This study found that while participants saw value in digital phenotyping technology, they also expressed data privacy concerns such as fears over data leakage. It is important to investigate whether results from this scoping study hold for school-aged adolescents and predictive mental health services based on a combination of data sources.

## Objective

Our study aimed to investigate adolescents’ interest in services that predict risk of mental health challenges and their preferences and attitudes towards sharing personal data for this purpose. We investigated young people’s attitudes towards sharing three types of data that have been investigated in predictive psychiatry research: biological data (eg, DNA),^10^ psychosocial data (eg, grades, psychological tests)^20^ and digital data (eg, Google search history, social media data).^1^ The study focused on adolescents aged 16–18 years enrolled in schools in the UK. We investigated the following questions:

1. Would young people want to take a predictive test for mental health challenges, and if so, which conditions and what motivates them to do so?
2. What types of data are young people willing to share for a predictive test, and how do they make this choice?
3. What are young people’s attitudes towards sharing data with schools and health systems for early identification of mental health challenges?

## Methods

### Design

The study was preregistered and all materials are available at https://osfio/cwjx4/. We recruited young people aged 16–18 years in schools in London and Oxford, UK. Parents were notified about the study, and participants provided informed consent before the session. A sample size of 80 participants was predetermined based on a previous report of young people’s attitudes towards testing for psychiatric conditions^13^ for comparability purposes. This sample size is considered large in qualitative research,^21^ allowing for a comprehensive understanding of adolescents’ perspectives.

### Procedure

Sessions took place at the participants’ schools, and in each session two randomly paired students were guided by a researcher (GP, JL, KW, LK or LG). Interview sessions started with a brief icebreaker. Each participant then independently completed a digital role-play titled *What Lies Ahead?* on a computer or laptop. The role-play simulated the experience of being offered a predictive mental health service and recorded participants’ choices. In a validation study, the scenario was shown to support immersion, authentic responses and reflective thinking.^22^

The role-play provided an entry point for a peer-to-peer interview, where participants took turns to ask and answer predefined questions to each other (drawing from a pile of flashcards) on their attitudes and preferences. Peer-led interviews can be more comfortable and engaging for young people than adult-led interviews and improve data quality.^23^

### Youth involvement

*What Lies Ahead?* was coproduced with the NeurOX Young People’s Advisory Group (YPAG), a group of 15–18 years olds who share an interest in ethics and mental health (https://begoodeie.com/ypag/). Through a series of group sessions, NeurOX YPAG members coproduced the role-play concept, gave input into the visuals and script, coled a pilot of the role-play concept and gave input into the interview guide. Part of the digital role-play was independently designed and produced by a group of five work experience students aged 16–18 years.

### Digital role-play

The digital role-play (approximately 5 min long) was presented from a first-person perspective (no avatar) and consisted of participants interacting with a staff member from ‘Future Forecast’, a predictive mental health company. The staff member presented the service and offered them the opportunity to sign up to learn their chances of facing mental health challenges in the future. Players were then asked to indicate, by clicking on tick boxes on a list of items on-screen: (a) what mental health challenges they would like to test for and (b) what data they would be happy to provide for the assessment, including digital, psychosocial and biological (see table 1 for items). Participants could select as many items as they wished and both lists included the option ‘none of the above’. Items included a range of mental health challenges and individual-level data sources used in psychiatric assessments and recent digital phenotyping research.^1 10 20^ All answers were recorded automatically via the digital role-play software.

**Table 1:** Role-play questions used to measure adolescents’ interest in mental health predictive testing, as well as preferences towards sharing personal data; created by the authors

Question	Items
1. Would you like to know your chances of facing the following challenges?	Depressed mood, attention and hyperactivity problems, disorganised thoughts and hearing voices, problems with anxiety, conduct problems, difficulties with memory and learning, addiction problems, problematic relationship with food, none of the above
2. What data would you be happy to provide for the assessment?	Digital data: Google search history, GPS and activity tracker information, information about the way I type on my phone (eg, typing speed), shopping history, social media data, text messages and voice conversations recorded from my phone, viewing history from video and music online platforms Biological data: Hormone levels, my genetic data, heart rate, general health records, a picture of my brain Psychosocial data: Lifestyle information from questionnaires, psychological tests, family mental health history, disciplinary records from my school, my grades Note: Items were presented ungrouped, in a randomly selected order; ‘none of the above’ was included as a final item

### Peer-led interview

The interview took approximately 30 min and questions prompted participants to justify and reflect on their choices during role-play. The interview guide (ie, flashcards) was standardised and also included questions about preferences and attitudes with regard to sharing data across systems (not covered in the role-play). These questions covered attitudes towards schools and public health services accessing health and school records, respectively, as well as social media data. While participants led the interview, the researcher mediated the interaction, offered clarifications and asked follow-up questions. Main questions are presented in table 2. Interviews were audio recorded and transcribed and names were replaced by pseudonyms.

**Table 2:** Main interview questions used to evaluate participants’ attitudes towards mental health predictive testing, sharing of personal data and cross-sectional data sharing; created by the authors

Topic	Interview questions
1. Taking a predictive test	Did you choose to take a predictive test or not? Why?
2. Data sharing	What types of data were you willing to share? Why these and not others? What types of data do you think are most/least private? What types of data do you think reveal most/least information about your mental health?
3. Cross-sectoral data sharing	If predictive testing for mental health challenges becomes part of routine check-ups… Would you be happy to share your school records with the health services for this purpose? Would you be happy to share health records with the school for this purpose? Would you be happy to share social media data with the school, and the health services for this purpose?

At the end of the session, participants filled in a brief demographic questionnaire. A short debriefing session followed, with the researcher emphasising that ‘Future Forecast’ is a hypothetical company and that predictive testing services as presented in the role-play are not currently available. Researchers also clarified any potential questions or concerns. After completing the first part of the interview, participants completed a second role-play task and interview. Data from this section is beyond the scope of the present paper and will not be reported.

### Data analysis

Participants’ interest in predictive testing for different conditions and willingness to share data (biological, psychosocial and digital and across sectors) was characterised using percentages. Data sharing preferences are reported for all participants, regardless of their expressed interest in taking a predictive test. There were missing data for interest in predictive testing (1/80) and in sharing data with health systems (2/80). All percentages refer to valid cases only. Interview transcripts were analysed using thematic analysis.^24^ We developed separate thematic frameworks for the sections of the interview that referred to interest in predictive testing, sharing personal data and cross-sectoral data sharing. The frameworks were based on the independent analysis of 25% of the transcripts by three different members of the research team; different interpretations of the data were discussed and consensus was achieved on the main categories to be coded. We then followed an iterative process of coding the rest of the transcripts and refining themes to best reflect the core ideas expressed by participants.

## Findings

### Participants

There were 80 participants in the study, aged from 16 to 18 years (Mage=16.9; SD=0.42), across six UK state schools. Participants were mainly females (59 females, 21 males) and were of different ethnicities (37.5% Asian or Asian British; 32.5% white; 20% black, African or black British; 10% mixed or other).

### Interest in predictive testing

**Figure 1:**
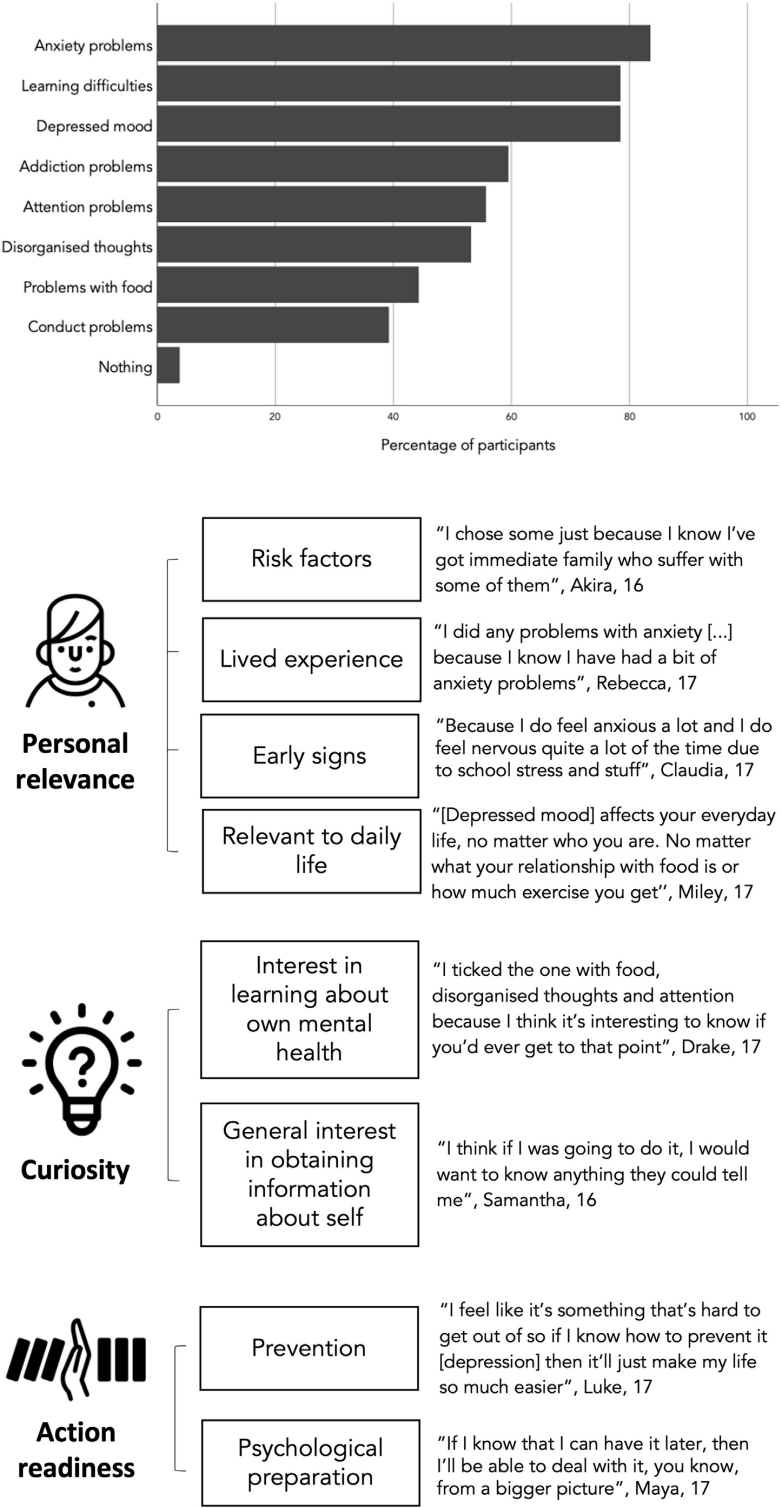
Percentage of participants interested in taking a predictive test for different mental health challenges (from digital role-play) and main reasons for taking a test (from interviews); created by the authors.

The majority of participants chose to take a predictive test for mental health challenges in the role-play (96.2%). Most participants chose to learn their risk for anxiety, learning difficulties and depression; the least chosen conditions were conduct problems and eating disorders (figure 1, top). Participants’ main motivations to take the test are summarised in figure 1, alongside illustrative quotes. About two-thirds of participants referred to the relevance of particular mental health challenges to their past and present lives. Relevant experiences included: awareness of being exposed to risk factors such as exam stress or family history of the condition; experience of similar challenges in the past; current experience of early signs of the condition or more generally, a feeling that certain conditions are relevant to their daily lives. An equally common theme was curiosity to learn about oneself and one’s future. This included expressing an interest in learning about one’s mental health and particular conditions, and more generally expressing that the more information they could obtain, the better. A third theme, mentioned by about a fifth of the interviewees, was the notion that being aware of one’s risk of developing a certain mental health problem later in life could support action readiness. This included the ability to take action to prevent challenges and (less frequently) the chance to ‘prepare for it’ from a psychological perspective. Only three participants expressed no interest in taking a test, either because they were ‘scared to find out’ or generally uninterested.

### Preferences and attitudes towards data sharing

**Figure 2:**
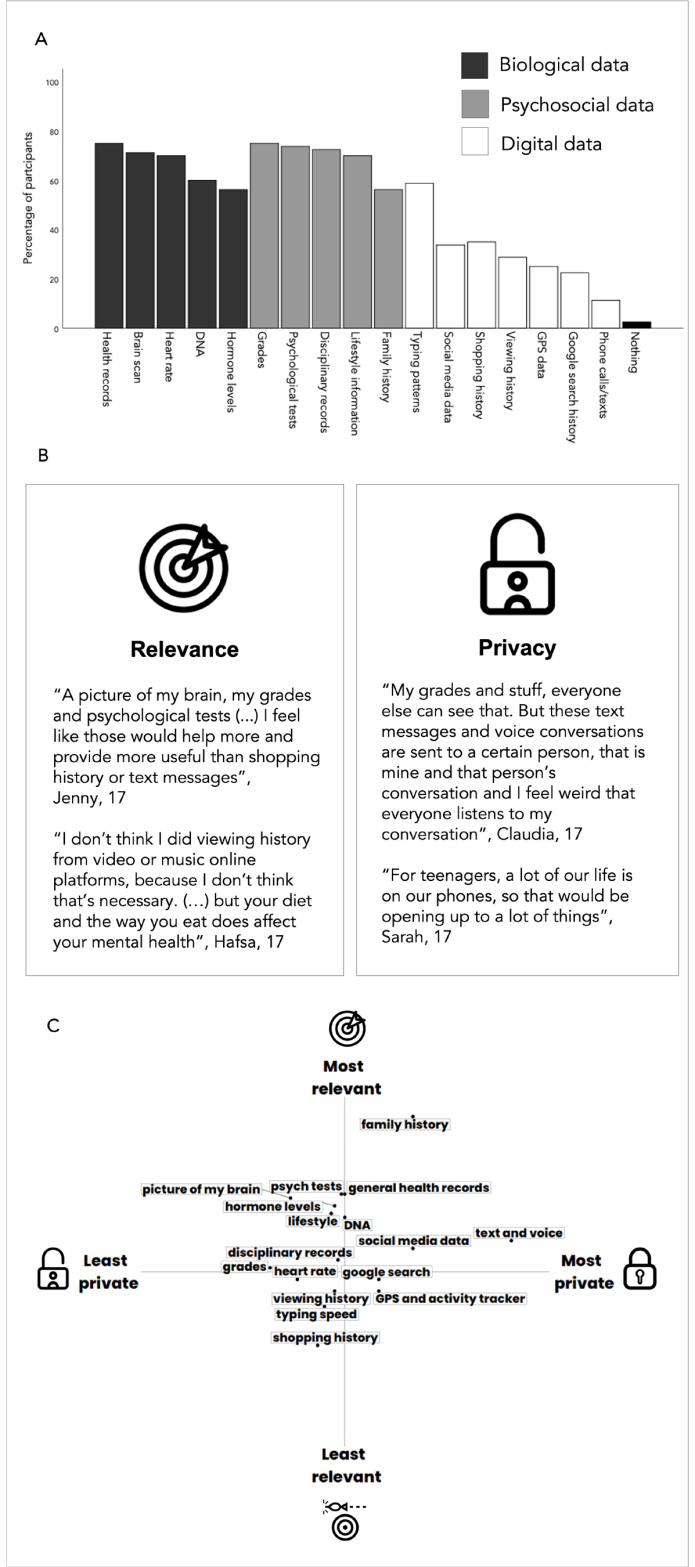
(A) Percentage of participants who shared different sources of personal data for mental health predictive test (from role-play). (B) Main reasons for sharing data (from interview). (C) Indicative two-dimensional configuration of data sources according to perceived privacy and relevance. Axes indicate the frequency with which the data source was referred to by participants when asked to comment on data sources most/least private and most/least revealing of their mental health (from interview); created by the authors.

Most participants were willing to share personal data for a predictive mental health test (97.5%). However, participants’ willingness to share personal information depended on data type (figure 2A). While most participants were willing to share biological data (66.5% on average) and psychosocial data (69.5% on average), only 30.7% of participants chose to share digital data sources. This average was even lower (26%) when excluding ‘typing patterns’. Two core themes were identified from participants’ justifications of data sharing choices: considerations around the relevance of the data source for predicting mental health issues and privacy concerns. Data sources perceived as more useful for the prediction were more likely to be shared than data considered less relevant. On the other hand, data sources considered more private were less likely to be shared than data considered less private (see figure 2B for quotes). Participants often made trade-off decisions, placing data sources across these two dimensions of relevance and privacy.

In figure 2C, we present an indicative, two-dimensional configuration of types of data participants referred to as most and least private and most and least relevant to a risk assessment. As illustrated, digital data sources such as Google search and GPS tended to be perceived as private; specific digital data sources perceived as not very private such as shopping history and typing speed tended to also be perceived as not as relevant. Psychosocial and biological data were perceived as generally relevant and not highly private. In addition to privacy and relevance, less common themes included access to the data source (‘I don’t even know my family’s mental health history’, Georgie, 17 years old) and curiosity about results arising from analysis of particular data sources (‘I search all weird stuff, so I thought it might be interesting to see if they have any links to anything’, Claire, 17 years old).

### Data sharing between systems

**Figure 3:**
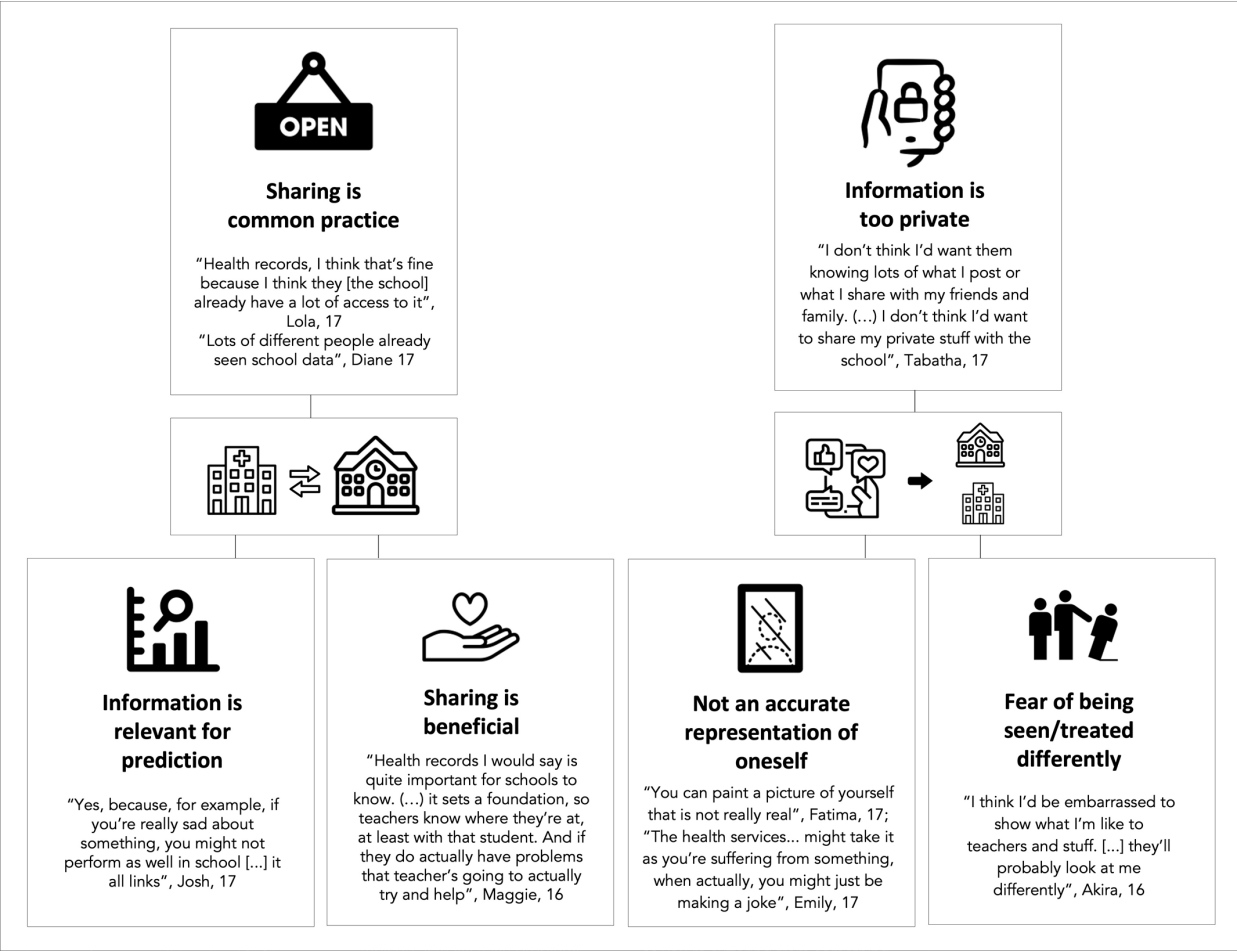
Main themes coded from participants’ arguments for sharing data between health and school systems (left) and for not sharing social media data with schools or health services to identify risk for mental health challenges; created by the authors.

The great majority of participants agreed with the health system accessing their school records (93.6%) and the school accessing health records (82.5%) for mental health predictive services. About a third of the participants indicated that sharing this information was already common practice (see figure 3 for quotes). Over a quarter explicitly mentioned that they considered cross-sectoral sharing to be beneficial to them and many referred to the information being relevant to the (early) detection of mental health challenges. The minority who refused to share health data with school typically argued that mental health screening was not the school’s responsibility (‘I feel like that’s more something that should happen in the medical, like the doctor world, that kind of thing. I don’t think it’s for school’, Pam, 17 years old).

When it comes to sharing social media data, however, while most participants (67.1%) agreed to share that information with the health system, only 30% were willing to share it with schools. Most participants raised privacy concerns due to the personal nature of this information and over a quarter indicated that predictions based on social media data might not be accurate, with some fearing being misinterpreted by an algorithm. A minority expressed concerns that they might be seen or treated differently by school staff specifically, should they know details about their personal life or mental health (see figure 3 for quotes). Willingness to share was largely justified based on the participant not posting information that is personal or secretive through social media platforms (‘Yes, I don’t mind. There’s nothing. Nothing there’, Miley, 17 years old), and a minority indicated the information could be relevant for the prediction (‘Actually yes, because some people, when they’re depressed, they go on social media, and they do those posts like, I’m really depressed, or whatever’, Steve, 18 years old). Further benefits were rarely mentioned, and those willing to share often emphasised only feeling comfortable sharing data already set as public (eg, open feed rather than direct messages).

## Discussion

Using a combination of digital role-play and peer-led interviews, our study found that most participants were interested in taking a mental health predictive test, especially for anxiety, learning difficulties and depression. Adolescents were motivated by the perceived relevance of mental health to themselves and their daily life; the opportunity to learn about themselves and the chance to engage in preventative measures. While participants valued predictive psychiatric services, they were selective with regard to what data sources to share for the assessment. Specifically, participants demonstrated significant reluctance to share digital footprints (in comparison to psychosocial and biological data), because of both concerns around privacy and—for some data sources—a perceived lack of utility. With regard to cross-sectoral data sharing, participants were positive about sharing school records with the health system and health records with schools; however, most were reluctant to share social media data with schools (but less so with the health system).

Participants’ high interest in taking a predictive psychiatric test aligns with research showing that adolescents value learning about themselves and their health.^25^ Participants’ differential interest in testing for specific conditions suggests a reasoned approach to healthcare decision-making. Notably, they were specifically motivated if they found the test personally relevant to them, for example, due to familial psychiatric history or emergent symptomatology. This is supported by prior research that documented high interest in learning about one’s risk among individuals at clinical high risk for psychosis^14^ and those with familial experience of depression.^26^ Participants’ caution around the privacy of digital data sources is also mirrored by research showing that adolescents attach high value to digital data privacy and protection.^18^

Participants’ considerations around the utility of different data sources resonate with concerns raised by researchers around the predictive value of psychiatric risk assessments.^9 10^ Researchers have also pointed to the related challenge of ‘algorithmic bias’, potentially leading to unfair or discriminatory results.^27 28^ While our participants did not mention biases directly, they did express concerns around the informative value of certain digital data sources and some feared being misrepresented by social media algorithms.

Results also indicated that young people base their decisions to share certain types of data not only on its usefulness and privacy but also on the end recipient of the shared data (eg, schools or health systems). These responses corroborate research highlighting the hesitancy of patients to share data when they have limited information or trust regarding its confidentiality.^29^ Unless the reasons for young people’s hesitancy to share digital data with schools are addressed and carefully considered by educational settings, the promise of social media-based algorithms to act as ‘virtual gatekeepers’^6^ will not be achieved.

Schools and providers of predictive psychiatric services must consider whether the relevance of different digital data sources for psychiatric assessments outweighs privacy concerns they might present to young people. This trade-off between privacy and relevance must be considered alongside other metrics such as the gravity of what is identified (eg, mild risk vs suicidal intent) and urgency to respond. Furthermore, in some cases, private information may be highly relevant but may not require a complex algorithm to accurately predict risk (eg, calls to a suicide prevention helpline). Young people, in particular those who have experienced mental health difficulties, should help inform which types of data sources are relevant and acceptable to assess risk, to whom and under what circumstances.

## Limitations

Our digital role-play successfully immersed participants in a simulated scenario and the peer-to-peer interview format facilitated self-initiated discussion and ethical reflection. However, it is possible that adolescents influenced each other’s responses, arriving at converging arguments, and that different decisions would be made in real life. Another potential limitation is that our role-play assumed adolescents were able to consent to mental health predictive testing, without the need for parental consent. Previous research indicates that parental consent requirement reduces adolescents' willingness to try digital mental health interventions,^30^ so it is possible that different decisions would be made should parental consent be required.

Our sample was meant to represent youth in the general population. The sample was diverse and included adolescents at risk or already experiencing mental health challenges. There was, however, an over-representation of females and possibly also of adolescents interested in mental health (who voluntarily signed up). Future research should help ascertain the extent to which results represent UK-wide adolescents and apply to particular demographic and risk groups.

## Clinical implications

Data from digital devices hold potential for detecting mental health challenges at an early stage, supporting prevention and early intervention in adolescent mental health. The demand for this technology has led to ‘function-creep’: the repurposing of software programmes to monitor young people’s mental health.^12^ Such repurposing has been especially prevalent within the educational context, where, for example, software used primarily for homework submission integrated additional capabilities for mental health monitoring (eg, Gaggle, Bark and Saasyan). Social media platforms have also integrated monitoring features (eg, Facebook monitoring suicide risk).^6^ In some cases, opting out of monitoring means opting out of the main service, leaving adolescents little choice on the use of their personal data.

As this study shows, young people see multiple benefits in predictive psychiatric services, but wish to have choice over which type of data is shared, with whom and for what purpose. Moreover, adolescents wish to know why different data sources are relevant for mental health prediction and how their privacy will be protected. Respecting adolescents’ need for privacy, transparency and choice in the age of digital phenotyping will be critical for responsible design and implementation of preventive psychiatric technologies and services in the future.

10.1136/ebmental-2021-300329.supp1 Supplementary data

## Data Availability

Data are available upon reasonable request. Data from the digital role-play are uploaded as supplementary information. Interview data are available upon reasonable request.
